# Molecular Mechanism of Antibody-Mediated Activation of β-galactosidase

**DOI:** 10.1016/j.str.2014.01.011

**Published:** 2014-04-08

**Authors:** Kutti R. Vinothkumar, Greg McMullan, Richard Henderson

**Affiliations:** 1 MRC Laboratory of Molecular Biology, Francis Crick Avenue, Cambridge CB2 0QH, UK

## Abstract

Binding of a single-chain Fv antibody to *Escherichia coli* β-galactosidase (β-gal) is known to stabilize the enzyme and activate several inactive point mutants, historically called antibody-mediated enzyme formation mutants. To understand the nature of this activation, we have determined by electron cryo-microscopy the structure of the complex between β-gal and the antibody scFv13R4. Our structure localizes the scFv13R4 binding site to the crevice between domains 1 and 3 in each β-gal subunit. The mutations that scFv13R4 counteracts are located between the antibody binding site and the active site of β-gal, at one end of the TIM-barrel that forms domain 3 where the substrate lactose is hydrolyzed. The mode of binding suggests how scFv stabilizes both the active site of β-gal and the tetrameric state.

## Highlights

• Antibodies can activate inactive mutant β-galactosidase enzymes
• Cryo-EM analysis reveals the structure of such an antibody complex
• One Fv antibody binds to each of four β-galactosidase subunits
• Activation occurs by internal stabilization within each subunit

## Graphical Abstract

**Figure:**
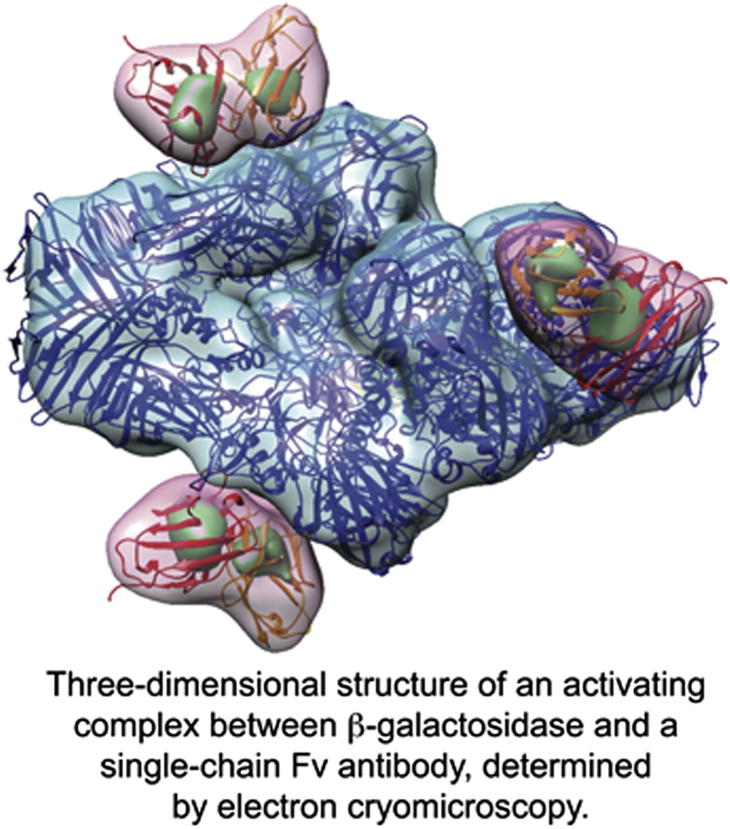

## Teaser

Certain inactive mutant β-galactosidases can be reactivated by anti-β-galactosidase antibodies. Vinothkumar et al. describe a cryoEM structure of the antibody:enzyme complex that shows how this activation occurs. Antibody binding indirectly stabilizes the active site and the intersubunit interface of the enzyme.

## Introduction

Recent advances in microscopes and detectors have resulted in single-particle electron cryo-microscopy (cryo-EM) becoming a powerful technique in structural biology. The ability to image macromolecules by cryo-EM and obtain a structure with a minimal amount of protein and without the need for crystals is a big advantage. We have been studying β-galactosidase (β-gal) by single-particle cryo-EM with the goal of reaching atomic resolution (Chen et al., 2013; Henderson et al., 2011). β-gal is a tetrameric enzyme, encoded by the *lacZ* gene of the lac operon, and it has been biochemically and structurally well studied (Dugdale et al., 2010; Juers et al., 2012). Early genetic studies had shown that coexpression or mixing of completely inactive polypeptides from certain pairs of mutant β-gal molecules could produce an active enzyme. This phenomenon, called α-complementation, is explained by the contribution of residues from two adjacent subunits in the tetramer to each active site, thus restoring the enzymatic activity when regions that include the nonmutated residues from adjacent subunits combine to form one active site. These genetic studies also identified mutants of β-gal that regain enzymatic activity upon incubation with particular antibodies, collectively called antibody-mediated enzyme formation (AMEF) mutants (Celada and Strom, 1972; Messer and Melchers, 1970). For example, the AMEF959 gene differs from the wild-type lacZ gene by one base substitution at codon 358: GAG (E) to AAG (K), thus changing a side chain with a negative charge into a side chain with a positive charge (Laden et al., 2002). This E358K mutant and several other AMEF mutants are point mutations. Two hypotheses have been put forward to explain their loss of activity (Laden et al., 2002). One hypothesis suggested that the enzyme remained tetrameric but that its active site was disrupted by the mutation and then restored by direct binding of the antibody. The second hypothesis attributed loss of activity to dissociation of the normally tetrameric enzyme into inactive dimers, with restoration of activity by binding of antibody to an epitope at the tetramer interface (de Macario et al., 1978). Binding of antibodies also increases the thermostability of wild-type β-gal (Melchers and Messer, 1970). Although both hypotheses have some experimental support, the evidence is not conclusive and may depend on the mutant in question.

Martineau et al. (1998) used the AMEF mutants to identify single-chain antibodies that can be expressed in the cytoplasm of *Escherichia coli* in a functional form in large quantities. A typical antibody has an intradomain disulfide bond, but when expressed in the reducing environment of the cytoplasm of *E. coli* the bond is not formed, frequently resulting in unfolded or unstable antibodies. Disulfide bonds linking the two β sheets in each antibody domain are normally formed in the endoplasmic reticulum or bacterial periplasm. To overcome this limitation, Martineau et al. (1998) developed a system for genetic selection of folded and functional antibody fragments in the cytoplasm using the AMEF mutants, with the goal of optimizing the expression, stability, and affinity of a single-chain Fv antibody domain. Starting with an Fv domain from a phage antibody library, they carried out four rounds of antibody mutation and selection using error-prone polymerase chain reaction in an *E. coli* strain coexpressing the mutant β-gal AMEF959 (E358K). By selecting for the ability to grow better in a galactose medium, they succeeded in obtaining a functional scFv fragment that expressed well and had a final dissociation constant of ∼2 μM (Martineau et al., 1998).

Using β-gal and scFv, we aimed to (1) observe and map by single-particle cryo-EM the location of the binding site for the 28 kDa scFv fragment and (2) explain how the antibody activates the enzyme. By mixing the enzyme and the antibody, we were able to form the β-gal:scFv complex and obtain images that gave an excellent 3D map. The map clearly shows the location of the scFv binding site and favors an explanation of antibody-mediated activation by a direct stabilization of the region of the protein where the AMEF mutations occur. This type of analysis of antibody-protein complexes is widely applicable for example to virus-neutralizing antibodies and other small structures of marginal stability, such as mutant p53 tumor suppressor proteins or HIV Env.

## Results

The dissociation constant between the scFv13R4 antibody domain and β-gal was estimated previously (Martineau et al., 1998) at 2 μM. At a β-gal concentration of ∼1 mg/ml, the enzyme active sites would be expected to be present at ∼9 μM. With the Fv concentration at ∼20 μM, the occupancy of each site, assuming four sites per tetramer, would be expected to be ∼90%. Assuming no cooperativity in binding, we would expect about half the complexes to be β-gal:(Fv)_4_ and half β-gal:(Fv)_3_, with a smaller number of dimeric and monomeric stoichiometries. The resulting maps are completely consistent with the expected stoichiometry and occupancy.

**Figure 1:**
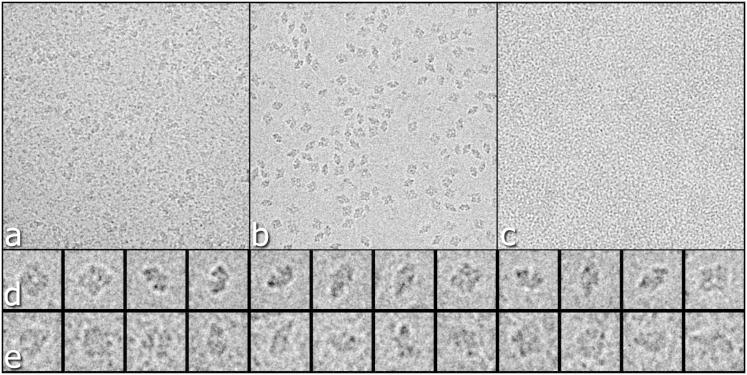
Typical Regions of Raw Micrographs and Selected Images of Single Particles Field of view in typical micrographs of (A) the β-gal:Fv complex (image 19.59.55), (B) β-gal without antibody (image 01.49.47), and (C) Fv antibody alone (image 13.35.50). We also show a gallery of some selected particles of (D) β-gal alone and (E) complex. Because of the background of free scFv antibodies with 28 kDa MW, the images of the complex look noisy. Scale, 3,100 Å edge in (A–C) and 260 Å for each panel in (D) and (E).

**Figure 2:**
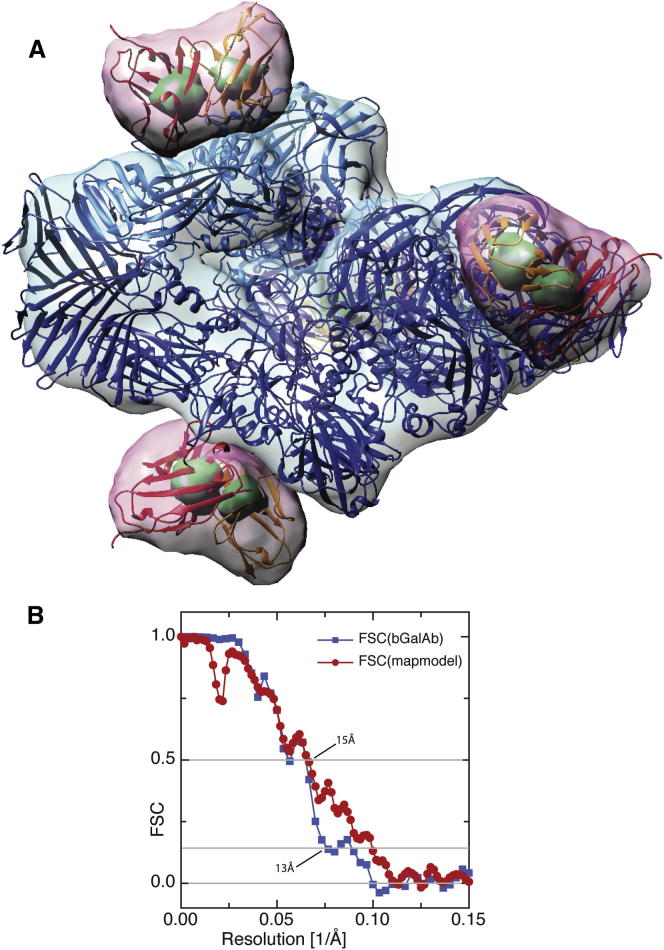
Surface-Contoured Structure of Complex between scFv13R4 and β-gal (A) Surface-contoured 3D map of β-gal alone (blue) and the difference map between β-gal and the β-gal:Fv complex (pink) with docked coordinates 1F4A of β-gal (Juers et al., 2000) and a typical Fv structure, taken from the 3A6B complex (Yokota et al., 2010) between hen egg lysozyme and a similar Fv. The maps were calculated using Frealign. The antibody domains are contoured at two different contour levels, showing the whole Fv domain at a lower contour level (pink) and the (just) resolved heavy and light chain densities at a higher contour level (green). (B) FSC plots for the β-gal:Fv complex. In blue squares, the FSC between experimental maps each calculated from half of the single-particle images shows a resolution at FSC 0.143 of ∼13 Å. In red circles, FSC between the 3D map and the density from the molecular model shows a resolution at FSC 0.5 of ∼15 Å. Figure S2 shows a similar 3D map and FSC plot for the structure computed using Relion.

Raw images of β-gal:scFv13 complex were much noisier than images of β-gal itself (Figure 1). Because we used an excess of Fv, there were six or seven free Fv molecules in the solution per β-gal:Fv complex, and the background in the spaces between the desired complexes was therefore filled with free Fv molecules that, at the defocus used, show up as small black dots. It was also possible to see the occasional GroEL structure (an impurity from the scFv purification), showing either the 7-fold top view or the striated edge view. Because these complexes at a molecular weight (MW) of ∼700 kDa are only slight larger than the combined β-gal (450 kDa) plus four Fv domains (4x28kDa), which has a combined MW of 560 kDa, we were careful to avoid picking any particles that looked like GroEL. A typical micrograph contained ∼60 good β-gal complexes, but possibly only one or two GroELs, so this was not a serious problem. Figure 1E shows some of the β-gal:Fv particles; these particles were are not visibly different from particles of β-gal alone, as shown in Figure 1D. Nevertheless, the single-particle 3D structure determination carried out using Frealign (Grigorieff, 2007) had no difficulty in orientation determination and 3D map refinement, resulting in the map shown in Figure 2A. A similar structure determination, calculated using Relion with its gold standard Fourier shell correlation (FSC)-weighting scheme (Scheres and Chen, 2012), is shown in Figure S2A (available online). The difference maps in Figure 2A and Figure S2A are contoured at two different levels, showing the overall outline of the Fv heavy and light chains at a low contour level (pink) and the two chains just resolved at a higher contour level (green).

**Figure 3:**
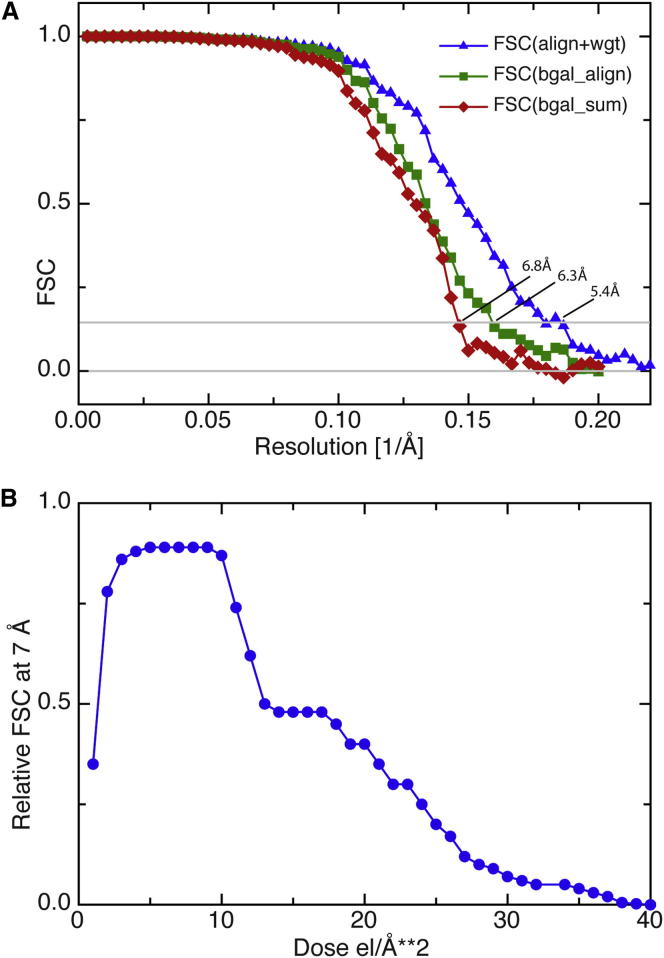
FSC Plots for the Structure of β-gal without Antibody (A) The FSC between two halves of the data for the structure of β-gal without antibody is shown for the simple sum of image frames (red diamonds), for the summed frames after alignment (green squares), and for the resolution-dependent weighted sums (blue triangles), showing resolutions of 6.8, 6.3, and 5.4 Å, respectively. (B) The weighting factor proportional to the FSC at ∼7 Å resolution is shown for 40 3D maps made from the first 40 single frames, each with a dose of 1 el/Å^2^. Figure S3 shows two additional plots: a tilt pair parameter plot to validate the overall procedure and high-resolution noise substitution to validate resolution.

After docking of the rigid-body models, the FSC between map and model is shown in Figure 2B, confirming the resolution estimate of ∼13 Å obtained internally from the Frealign run. The 3D map of β-gal alone (not shown), due to the lower background in the absence of Fv, the greater number of particles, and the use of subframe alignment and resolution-dependent weighting, had a resolution of 5.4 Å (Figure 3), a value approximately double that of the β-gal:Fv complex. This allowed more accurate magnification determination and docking of the β-gal atomic model, resulting in more accurate identification of the residues involved in Fv binding.

Residues involved in the contact with antibody and therefore in mediating the rescue and stabilization of the AMEF mutated β-gal are listed in Table 1. Although the β-gal:Fv map was good enough to resolve the two Fv domains, it was not good enough to identify which domain represented the heavy chain and which represented the light chain. Nevertheless, it appears that we have fortuitously chosen the correct locations for heavy and light chains. As seen in Table 1, there are extensive contacts between all the complementarity determining regions (CDRs) of the heavy chain and domain 3 of β-gal, whereas there are minimal contacts between the light chain and domain 1 of β-gal, consisting of only a single possible contact between the light chain CDR2 and domain 1 of β-gal. In the earlier mutagenesis selection procedure (Martineau et al., 1998), six mutations throughout the heavy chain and only one mutation in CRD2 of the light chain contributed to the increased affinity. There is therefore excellent correlation between the most extensive β-gal:Fv contact regions seen in our map and the most extensive sites of the earlier selected mutations (Martineau et al., 1998).

## Discussion

**Figure 4:**
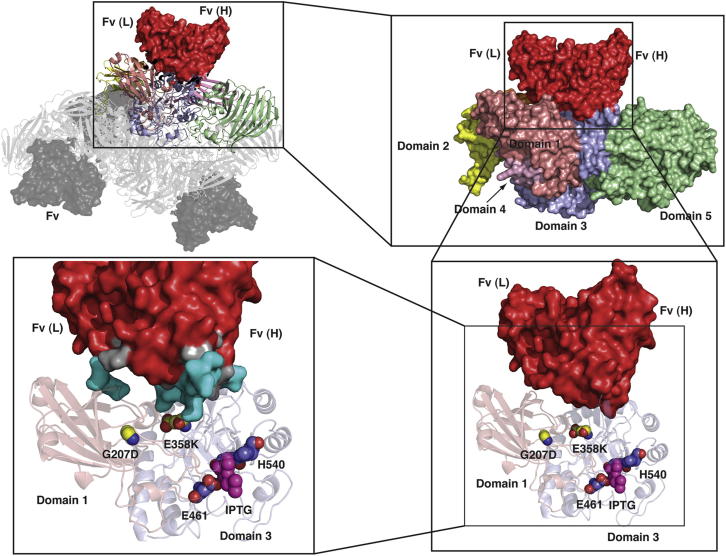
Geometrical Relationship between the Fv Antibody and Key Residues in the Enzyme Each Fv antibody binds to an extended surface of one β-gal subunit that is made up of part of the N-terminal domain 1 and part of domain 3, the core β-barrel catalytic domain that lies at the heart of each of the β-gal subunits. The illustration shows the entire structure (top left); one β-gal subunit with each of the five domains in different colors (top right); and parts of domains 1 and 3 from one β-gal subunit (bottom right and bottom left), rotated ∼30° to the left, showing the region that is in contact with the (red) Fv. The amino acid E358K (yellow) that is mutated in AMEF959, the amino acid G207D (yellow) that is mutated in AMEF645, and the two active-site residues E461 and H540 are labeled. A purple atomic model of IPTG (from PDB 3DYO) shows the location of the substrate binding site. The most extensive contacts, shown at bottom left and listed in Table 1, are between the Fv heavy chain and domain 3 of β-gal, with the antibody surface shown in dark gray and the β-gal surface in cyan. Figure S4 shows a stereo plot of the same region.

One of the hypotheses advanced originally, to explain the loss of activity in mutant AMEF β-galactosidases and subsequent regain of activity through AMEF, was that mutated enzymes were dimers and that the antibodies bound to and stabilized an epitope formed at the interface between two dimers (de Macario et al., 1978), thus stimulating tetramer formation. In Figure 4, we show the geometrical relationship between each Fv binding site and each subunit of β-gal. There is contact between one Fv and only one of the four β-gal monomers, with the nearest distance from any atom in the Fv to any atom in the next-nearest β-gal subunit being 28 Å. Because there is no contact between scFv13R4 and any neighboring β-gal subunit, Fv binding can therefore directly stabilize only the β-gal subunit to which it binds. The locations of the AMEF mutation E358K used for selection of scFv13R4, and of another mutant G207D that is also activated by scFv13R4, are shown in Figure 4. The mutations E358K and G207D are 16.5 and 22 Å distant from the nearest atom of scFv13R4, respectively. The center of a typical bound substrate or inhibitor (e.g., isopropyl-thio-galactoside [IPTG], shown in Figure 4) is located ∼26 Å distant from the nearest atom of scFv13R4. ScFv13R4 binds to a crevice in each subunit between the N-terminal domain 1 of β-gal and the catalytic core domain 3. Because mutation E358K is in domain 3, and G207D is in domain 1, scFv13R4 binding can directly stabilize the region of the protein near both mutations. Our structure thus seems to indicate that it is the direct stabilization of the active site within each subunit by scFv13R4 that restores the activity. The tendency for dissociation of tetramers into dimers in AMEF mutants, and the subsequent stabilization of the tetramer by antibodies, is likely to be an indirect consequence of the mutation, because the epitope bound by scFv13R4 is located well away from the dimer:dimer interface. The structure of β-gal shows that a loop of ∼16 amino acids from a neighboring monomer extends across the active site and also that several residues from domain 3 form part of the dimer:dimer interface. Mutations distant from the dimerization interface can thus result in destabilization of the tetramer.

It seems likely that the local region of the protein surface to which the antibody binds will be held in a more rigid conformation than in the antibody-free state. This rigidification presumably can then extend through the structure to an extent that will reach the sites of the destabilizing mutations (16 Å distant), and subsequently to both the active site (26 Å distant) and even to the subunit:subunit interfaces (28 Å distant). Thus, there may not be a clear yes or no answer to the question of whether the antibody-mediated enzyme activation occurs by a mechanism that is localized entirely to within the subunit to which it binds or by a mechanism that indirectly increases the tetramer stability. It is certain however that antibody binding does not primarily stabilize the tetramer interface, but rather affects the entire protein. In general, it may not be easy to dissect the relative importance of these two possible ways of stabilizing the structure of an enzyme or other multisubunit or multidomain protein by antibody binding. Clearly, part of the Fv binding energy is being used to push the structure into a conformation that favors both catalytic activity and tetramerization, particularly when the magnitude of this binding energy is increased by powerful in vitro mutagenesis followed by selection for growth that requires enzyme activity.

The conclusions in this paper were anticipated by Laden et al. (2002), even though they had no knowledge of the location of the antibody binding. They explicitly suggested because the two mutations might play a role in stabilizing the conformation of the active site and because the locations of the mutations were closer to the active site than to the dimer interface, that the second mechanism (i.e., internal stabilization within each subunit) was more likely to be correct. They also made the argument that because the antibody is specific for the native conformation of the enzyme, it should help constrain the enzyme to its native, active state. Both these insights are confirmed and extended by the present results.

Small single-domain antibodies, such as that studied in this paper, are valuable in research, diagnostics, and therapy (Holliger and Hudson, 2005). Understanding how an antibody binds to a particular protein is essential for understanding the nature of the epitope it recognizes. This understanding will help in the evolution of higher affinity antibodies for better treatment of disease. Growing crystals of protein-antibody complexes for analysis by X-ray crystallography can be tricky, whereas single-particle cryo-EM, as shown here with β-gal bound to scFV, can be relatively straightforward. Even at moderate resolution, it is possible to understand the biology behind the interactions. Analysis of such protein complexes by cryo-EM may soon extend beyond basic research to become a tool for rational drug development by the pharmaceutical industry.

## Experimental Procedures

### Expression and Purification of scFv13

The gene encoding scFv13R4 was expressed as described previously (Martineau et al., 1998) in a pET 16b vector in *E. coli* strain PM12 (BL21 (DE3) pLysS with a *lacZ*:Tn5 mutation. A single colony was inoculated into 6 ml of Luria broth (LB) medium with ampicillin and chloramphenicol and grown for 10 hr at 37°C. Then, 1 ml of this culture was diluted into 50 ml of LB medium and grown overnight at 37°C. Cells (25 ml) were then diluted into 1 l of 2xYT and grown at 37°C at 200 rpm. Expression of scFv13R4 was induced at optical density 600 of ∼0.5 with 0.4 mM IPTG, and then cells were again grown overnight at 24°C. Cells were harvested and lysed with an Emulsiflex homogenizer (Avestin), and unbroken cells were removed by centrifugation at 5,000 × *g* for 20 min. The supernatant was loaded onto a nickel-nitrilotriacetic acid column (QIAGEN) pre-equilibrated with 25 mM Tris (pH 8.0) and 0.2 M NaCl (buffer A). The column was washed with buffer A and buffer A with 10 mM imidazole. scFv13R4 was eluted with 0.2 M imidazole in buffer A. Protein fractions at a concentration of ∼2 mg/ml were snap-frozen in liquid nitrogen and stored at −80°C.

### Specimen Preparation for Cryo-EM and Imaging

Commercially available β-gal (Sigma catalog no. G3153) was used. For complex formation, β-gal was mixed with scFv13R4 in a 1:10 stoichiometric ratio (i.e., 2.5-fold excess) and incubated in ice for 1 hr. Then, 3 μl of β-gal, its complex with scFv13R4, or scFv13R4 alone was applied to Quantifoil grids (1.2 μm holes), blotted for 5 s, and plunge-frozen in liquid ethane at near liquid nitrogen temperature, using an environmental plunge-freeze apparatus (Bellare et al., 1988). Grids were transferred into a Polara G2 electron microscope (FEI), and low-dose images were recorded on a Falcon II CMOS direct electron detector (FEI) at 300 keV and 80,240× magnification (59,000× nominal), with specimen temperature at −186°C. Images were recorded with a range of defocus values between 1.5 and 5.0 μm, and typical exposures times of 4 s. The pixel size is 14 μm on the Falcon detector, translating into a sampling rate of ∼1.75 Å/pixel. Nominal pixel sizes required later refinement by up to 3% against an atomic model from X-ray crystallography (Dugdale et al., 2010) to obtain the correct magnification.

### Image Processing and 3D Reconstruction

We selected 49 images out of a total of 52 recorded based on good ice thickness and reasonable particle visibility. A total of 2,965 particles were picked by hand using Ximdisp (Smith, 1999). CTFs were estimated using CTFFIND3 (Mindell and Grigorieff, 2003), boxed particle densities were floated and normalized using BOXIMAGE (Crowther et al., 1996), and 3D maps were calculated using Frealign (Grigorieff, 2007). Part of a typical micrograph is shown in Figure 1A. Comparison with a similar micrograph of β-gal without scFv, shown in Figure 1B, shows that the images of the complex look much noisier because the free 28 kDa scFv particles show up as a background of smaller black dots (Figure 1C).

A starting reference map was calculated from the atomic coordinates of *E. coli* β-gal structure (Dugdale et al., 2010), using Protein Data Bank (PDB) coordinates 1F4A (Juers et al., 2000). A rough correction for the fact that the real specimen is embedded in a solvent (amorphous ice) with a density of ∼80% of that of protein was applied by subtracting from a map calculated from atomic coordinates in vacuo; another map with a B-factor of 2,000; and a scale factor of 0.8. This solvent correction affects only the low-resolution structure: its effect falls to about a third at 20 Å and is negligible by 10 Å. The 80% factor is a rough approximation for the amount of electron scattering by ice compared with protein, although a slightly lower value of 72% was estimated previously (Rosenthal and Henderson, 2003). Note that we could equally well have used a good experimental cryo-EM map as the starting reference (Henderson et al., 2011).

The particle images were binned 2 × 2 to give a nominal pixel size of 3.5Å, corresponding to a potential resolution at Nyquist of 7.0 Å. A high-resolution cut-off of 14 Å was used during refinement of the particle position and orientation parameters to avoid the possibility of overfitting (Chen et al., 2013). The density representing the antibody domain started to appear in the first cycle of determination of particle orientation and position. The map did not improve after 12 cycles, showing a resolution of ∼13 Å at an FSC value of 0.143. The density representing only the antibody domains was determined by subtracting a 3D map of β-gal from the 3D map of the β-gal:Fv complex, with both maps on the same scale and with no B-factor sharpening (Rosenthal and Henderson, 2003). The antibody domains had a peak density of approximately eight times the root mean square density in the difference map and more than 90% of the peak density in the β-gal region of the map, so we estimate the occupancy to be about 90%. Later calculation of FSC between the map and an atomic model of the β-gal:Fv complex with variable Fv occupancy also gave best agreement with 90% occupancy.

### Rigid-Body Docking of Atomic Models

Molecular models representing β-gal (Juers et al., 2000) and a typical Fv domain, HyHEL-10 (Yokota et al., 2010), were docked into the 3D density map using Chimera (Pettersen et al., 2004), and the resulting structure is shown in Figure 2. The Fv from the lysozyme:HyHEL-10 complex was chosen because the original Fab complex (Padlan et al., 1989) was one of the first-known structures of an antibody bound to its macromolecular antigen. The variable domains also have ∼40% sequence identity with scFv13R4, with only small deletions in four of the six complementarity determining regions (see Figure S1). The PDB accession numbers for the β-gal and the Fv coordinates used are 1F4A and 3A6B, respectively. At an appropriate contour level, the two domains of the Fv structure could be resolved, but even after sharpening it was not possible to assign which of the two variable domains within the Fv structure corresponded to the light chain and heavy chain sequences.

### Improved Cryo-EM Structure of β-gal

We also include in this paper a description of the method used to produce the cryo-EM map of the uncomplexed β-gal. Eighty-nine images of β-gal alone were recorded with simultaneous acquisition of individual movie frames in the rolling-shutter mode of data acquisition by the Falcon CMOS detector. Typically, exposures were recorded as full-resolution movies that contained up to 80 frames (at 18 Hz frame rate). The exposures were saved as both single images that consisted of the sum of all the frames and as a full-resolution movie. Exposures varied from 2 to 5 s (34–80 electrons [el]/Å^2^). Using Ximdisp, 43,000 single particles were manually picked. Movement of these particles during the exposure was tracked using a noise-weighted cross-correlation. In this cross-correlation, a 256 × 256 pixel box was placed about each particle, and the motion of a particle relative to the self-consistent average particle calculated. The cross-correlation was calculated using Fourier components between 1/200 and 1/25 Å^−1^ and care was taken to avoid the residual fixed pattern signal from the detector. Because β-gal molecules are relatively small, the signal from individual frames was too weak to reliably determine a cross-correlation peak. A reliable peak could however be determined by using running averages over 11 frames. These calculations indicated that neighboring particles moved together, so final movements were determined from the combined cross-correlation estimate from the particle and all particles within a radius of 1,000 Å from the center of the particle. The nature of the particles is not important for movement determination, so additional particles were selected from the images using an automated particle picker. With the additional signal from the neighboring particles, the number of frames in the running average was reduced to five. The resolution of the 3D map calculated from single-particle images obtained after this alignment improved from 6.8 to 6.3 Å, as shown in Figure 3A). It was also possible to calculate 3D maps from single frames of all the particles and to examine the information content of each of the frames at different spatial frequencies from the FSC. Cross-correlation at ∼7 Å resolution between the 3D map obtained from each frame and the map from the summed frames is shown in Figure 3B. It shows that the first three frames, with each frame having an exposure of 1 el/Å^2^, had lower information content at ∼7 Å resolution than frames 4–9, which had the most information. The information content of subsequent frames (i.e., frames 10–40) appeared to follow the decay expected from radiation damage at ∼7 Å resolution and liquid nitrogen temperature that was observed previously by following the decay of electron diffraction patterns from 2D crystals of bacteriorhodopsin (Stark et al., 1996). By applying resolution-dependent weighting to each frame according to the information content, the resolution of the final 3D map was further improved to ∼5.4 Å, as shown in Figure 3A. The resolution-dependent weighting was applied using TWOFILE (Crowther et al., 1996) to multiply the Fourier transform of each frame of the aligned images of each particle by a resolution-dependent number derived from the FSC curve for the 3D maps from the individual frames described above. The frame- and resolution-dependent multipliers were produced by a program called MAKE_RESOL_WEIGHTS that is available on request. The weighted Fourier transforms of the individual frames were then back-transformed and summed to produce the optimized single-particle images. Using this procedure, the later frames, for example from 40 to 80 el/Å^2^, contributed to this final map only at lower resolution.

### Map and Model Deposition

The maps of the β-gal:scFv complex, β-gal alone, and the antibody difference map have been deposited in Electron Microscopy Data Bank with accession number EMD-2548. The model of the β-gal:scFv complex has been deposited in PDB with accession number 4CKD.

## Figures and Tables

**Table 1:** Possible Contacts between scFv13R4 (*HyHEL-10 docked*) and β-gal

Antibody Residues	β-gal Residues
Light chain S56(*S56*) (CDR2)	N55, E57 domain 1
Heavy chain E1(*D1*) (N-term)	E57 domain 1
Heavy chain S30(*T30*) (CDR1)	I576, K577, Y578 domain 3
Heavy chain N31, S33(*S31, Y33*) (CDR1)	L362 domain 3
Heavy chain S33(*Y33*) (CDR1)	D610 domain 3
Heavy chain S53(*Y53*) (CDR2)	P361, H363, A609 domain 3
Heavy chain S54(*S54*) (CDR2)	T612 domain 3
Heavy chain R71(*R71*) (outer loop)	P584 domain 3
Heavy chain N73(*T73*) (outer loop)	D579, G582, N583 domain 3
Heavy chain A74(*S74*) (outer loop)	E580, N581 domain 3
Heavy chain S95, S96(*W98, D99*) (CDR3)	Q370, domain 3

Residue numbering in the first column, such as S56, refers to the amino acid sequence of scFv13R4 from Martineau et al. (1998) and, in parentheses italicized, from the homologous docked lysozyme:HyHEL-10 Fv structure, with PDB accession number 3A6B.
