# Supplementary material for: Molecular Mechanism of Antibody-Mediated Activation of β-galactosidase
Source: Structure. 2014 Apr 8;22(4):621–7. doi: 10.1016/j.str.2014.01.011 (PMC3988998; doi:10.1016/j.str.2014.01.011)
Supplement: Document S1. Figures S1–S4 [file mmc1.pdf]

Structure, Volume *22*

Supplemental Information

Molecular Mechanism of Antibody-Mediated Activation of β-galactosidase

Kutti R. Vinothkumar, Greg McMullan, and Richard Henderson

Supplementary Information SI.Fig.1 (associated with Table 1) – Sequence alignment of scFv13R4 with HyHEL-10

scFv13R4 sequence, heavy chain first, light chain second
3A6B HYHEL-10 from PDB

```
   1                              cdr1              48
 MAEVQLVESGGSLVKPGGSLRLSCAASGFTFSNYSMNWVRQAPGKGLEWI
   DVQLQESGPSLVKPSQTLSLTCSVTGDSITSDYWSWIRKFPGNRLEYM
   1                                                48

49   a        cdr2                    abc           94
 SSISGSSRYIYYADFVKGRFTISRDNATNSLYLQMNSLRAEDTAVYYCVR
 GYVS-YSGSTYYNPSLKSRISITRDTSKNQYYLDLNSVTTEDTATYYCAN
49                                                  97
      cdr3
95     abcd             113           linker
 SSITIFGGGMDVWGRGTLVTVSS---------GGGGSGGGGSGGGGS---
 WDG-------DYWGQGTLVTVSAA
98                 114
                              cdr1
 1                            abc                   47
 QSVLTQPAS-VSGSPGQSITISGAGTSSDVGGYNYVSWYQQHPGKAPKLM
 DIVLTQSPATLSVTPGNSVSLSCRASQS-IGNDLH--WYQQKSHESPRLL
 1                                                  47

48   cdr2                                   cdr3   a96
 IYEDSKRPSGVSNRFSGSKSGNTASLTISGLQAEDEADYYCSSYTTRSTR
 IKYASQSISGIPSRFSGSGSGTDFTLSINSVETEDFGMYFCQQSNSWP-Y
48                                                  96

97        a   110      myc tag     his tag
 VFGGGTKLAVLGAAA      EQKLISEEDLNGAAHHHHHH
 TFGGGTKLEIK
97        107
```

Complementarity determining regions (CDRs) are shown in bold type with cyan highlighting. Mutations introduced by Martineau et al (1998) between scFv13 and scFv13R4 are in red. Most loops are of similar length except for the heavy chain CDR3, which has 7 extra residues but is not involved in contact with β-gal.

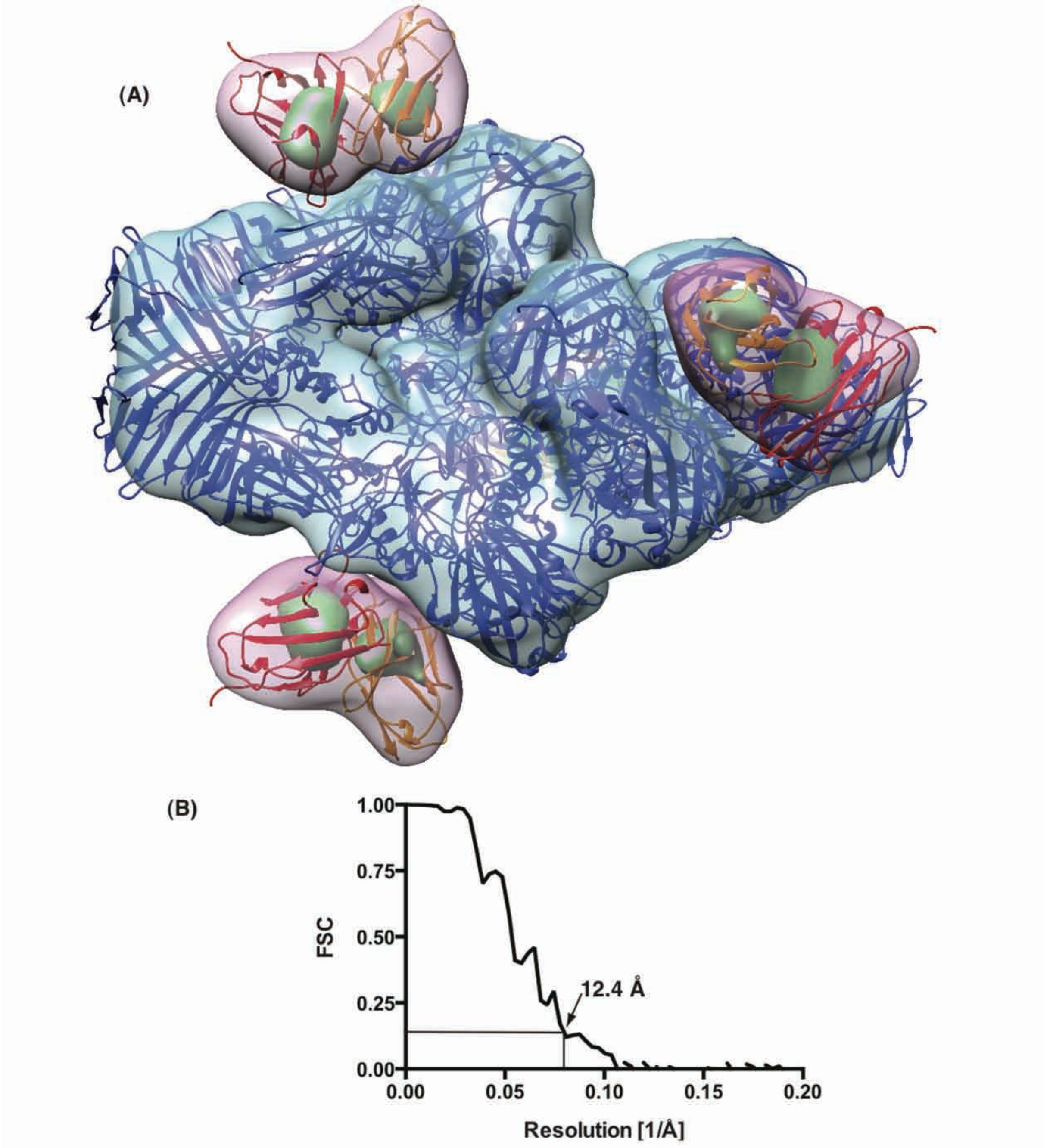

SI.Fig.2 (related to Fig.2). Validation of structure determination by use of an independent procedure for structure determination of the β-gal:Fv complex, namely Relion using gold-standard FSC weighting (Scheres and Chen, 2012). (a) shows, using the same scheme as in Fig.2, a map of the β-gal:Fv complex calculated completely independently. (b) shows the final Relion gold-standard FSC plot, showing a similar resolution (12.4 Å instead of 13.1 Å). The starting model was a 60 Å resolution, low-pass filtered map of β-galactosidase without the antibody.

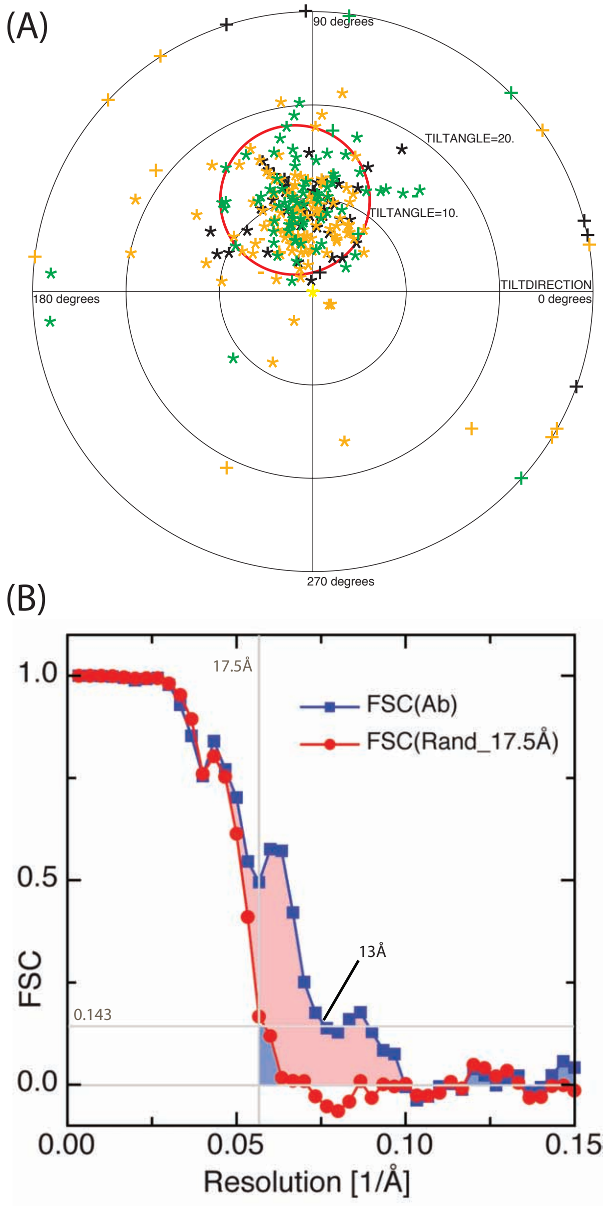

SI.Fig.3 (related to Fig.3). Although tilt-pair validation and high-resolution noise substitution have been used to validate previous maps of β-galactosidase from similar images (Chen et al., 2013; Henderson et al., 2011), we include here for completeness (a) a tilt pair parameter plot (TPPP) using the new β-gal map, and (b) an FSC plot of the high-resolution noise substituted β-gal:Fv complex map, using 17.5Å resolution for noise substitution. Tilt pairs were not collected for the antibody complex, only for β-gal alone. The TPPP clustering and the pink shaded area in the HR-noise substituted plot confirms the estimated resolution.

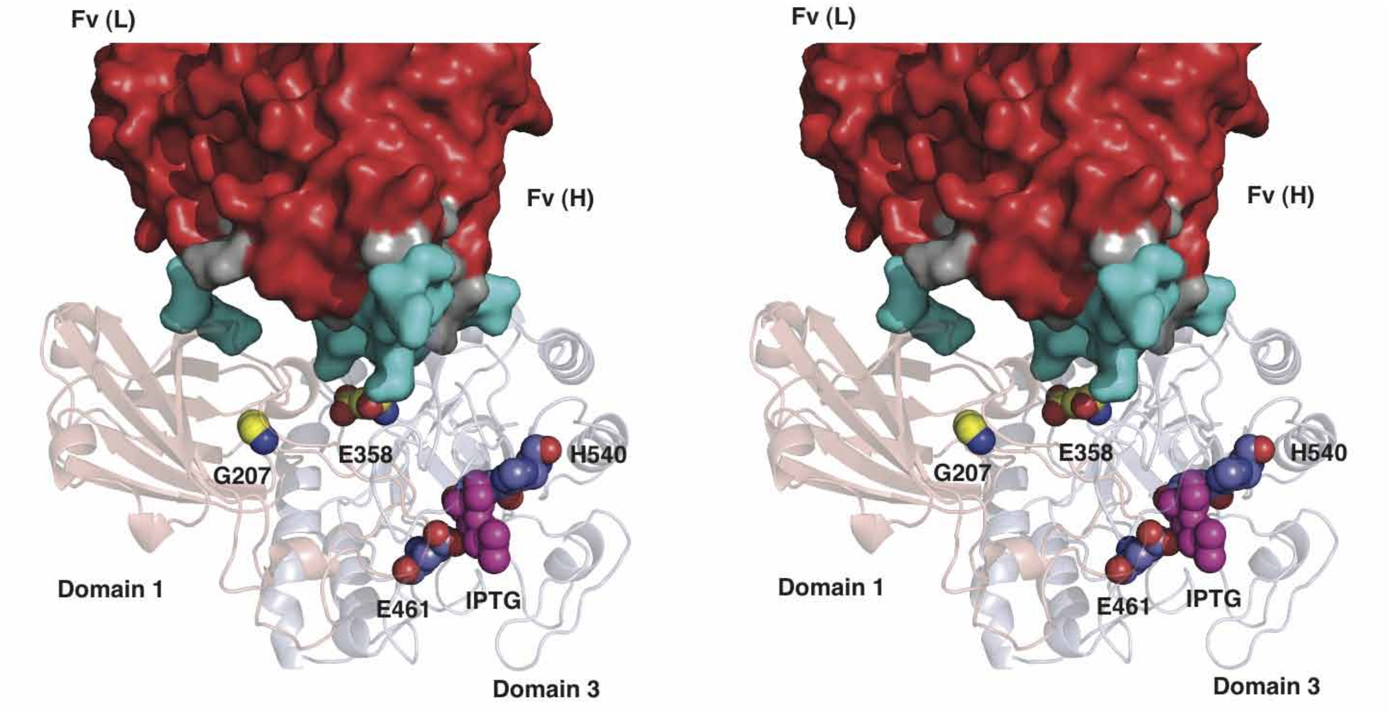

SI.Fig.4 (related to Fig.4). Stereo pair image corresponding to the last panel of Fig.4, showing the amino acid E358K (yellow) that is mutated in AMEF959 and the amino acid G207D (yellow) that is mutated in AMEF645. Two active site residues (E461 and H540) are labeled, with a purple atomic model of IPTG showing the substrate binding site. The contact surfaces, between the antibody in dark grey and β-gal in cyan, is shown.
